# A case of subacute infective endocarditis and blood access infection caused by *Enterococcus durans*

**DOI:** 10.1186/1471-2334-13-594

**Published:** 2013-12-17

**Authors:** Tsuneaki Kenzaka, Noriko Takamura, Ayako Kumabe, Koichi Takeda

**Affiliations:** 1 Division of General Medicine, Center for Community Medicine, Jichi Medical University School of Medicine, Shimotsuke, Japan

**Keywords:** *Enterococcus durans*, Infective endocarditis, Blood access infection, Infective aneurysm

## Abstract

**Background:**

Infection by *Enterococcus durans* (*E. durans*) is very rare; reported cases are often preceded by therapy or an immunosuppressed state, including infective endocarditis, urinary tract infection, or wound infection. A few reported cases of infective endocarditis exist, with no reports describing involvement of blood access infection.

**Case presentation:**

The patient is an 83-year-old man who had been undergoing hemodialysis for 8 years due to renal failure caused by diabetic nephropathy. He developed infective endocarditis and blood access infection/infective aneurysm due to *Enterococcus durans*; these conditions were treated with the antibiotic regimen of ampicillin + gentamicin. There have been only a few reported cases of infective endocarditis caused by *E. durans*, and to our knowledge, this is the first report of blood access infection.

**Conclusions:**

We have experienced a case of concurrent infective endocarditis and blood access infection/infective aneurysm caused by *E. durans*. This is the world’s first reported case of blood access infection/infective aneurysm by *E. durans*.

## Background

Infection by *Enterococcus durans* (*E. durans*) is very rare; reported cases are often preceded by therapy or an immunosuppressed state, including infective endocarditis, urinary tract infection, or wound infection. A few reported cases of infective endocarditis exist, with no reports describing involvement of blood access infection. We report a case of infective endocarditis and blood access infection/infective aneurysm caused by *E. durans*.

## Case presentation

The patient is an 83-year-old man with a 50-year medical history of diabetes; he had been undergoing hemodialysis for 8 years due to renal failure caused by diabetic nephropathy. After repeated shunt failure, the superficialized right brachial artery had been used for blood access for the past 2 years. About 4 months prior to admission, an occasional fever with temperature of 38°C would repeatedly occur after hemodialysis. About 1 month prior to admission, the frequency of fever increased, and levofloxacin and cefotiam were administered but yielded no improvement; the day of dialysis would be followed by an overnight fever of about 38°C accompanied by chills and joint pain. From about 2 weeks prior to presentation, general malaise and loss of appetite was observed; hence, the patient was hospitalized 6 days before the current medical consult. Chest and pelvic CT examination was performed; however, the cause of his fever and chills could not be identified. *Enterococcus* was detected in a blood culture performed before and after hemodialysis on admission, and the patient was transferred to our hospital for examination and treatment of bacteremia.

Upon admission, blood pressure of the patient was 145/68 mmHg, pulse was 64 beats/min, breathing rate was 20 breaths/min, and body temperature was 37°C. During physical examination, a Levine III/VI systolic ejection murmur was heard in the aortic valve area. No Osler’s nodes or Janeway lesions were observed, and blood from the dialysis puncture site was free of abnormalities.

The laboratory findings were as follows: a white blood cell count of 6,900/μL (neutrophils, 78%); hemoglobin, 10.7 g/dL; platelets, 117,000/μL; C-reactive protein, 3.4 mg/dL; total bilirubin, 0.5 mg/dL; aspartate aminotransferase, 23 IU/L; alanine aminotransferase, 7 IU/L; lactic dehydrogenase, 137 IU/L; gamma-glutamyl transpeptidase, 16 IU/L; creatinine phosphokinase, 34 IU/L; blood urea nitrogen, 26 mg/dL; creatinine, 6.22 mg/dL; Na, 139 mEq/L; K, 4.0 mEq/L; Cl, 101 mEq/L; blood glucose, 148 mg/dL; and hemoglobin A1c (NGSP), 6.0%.

**Figure 1:**
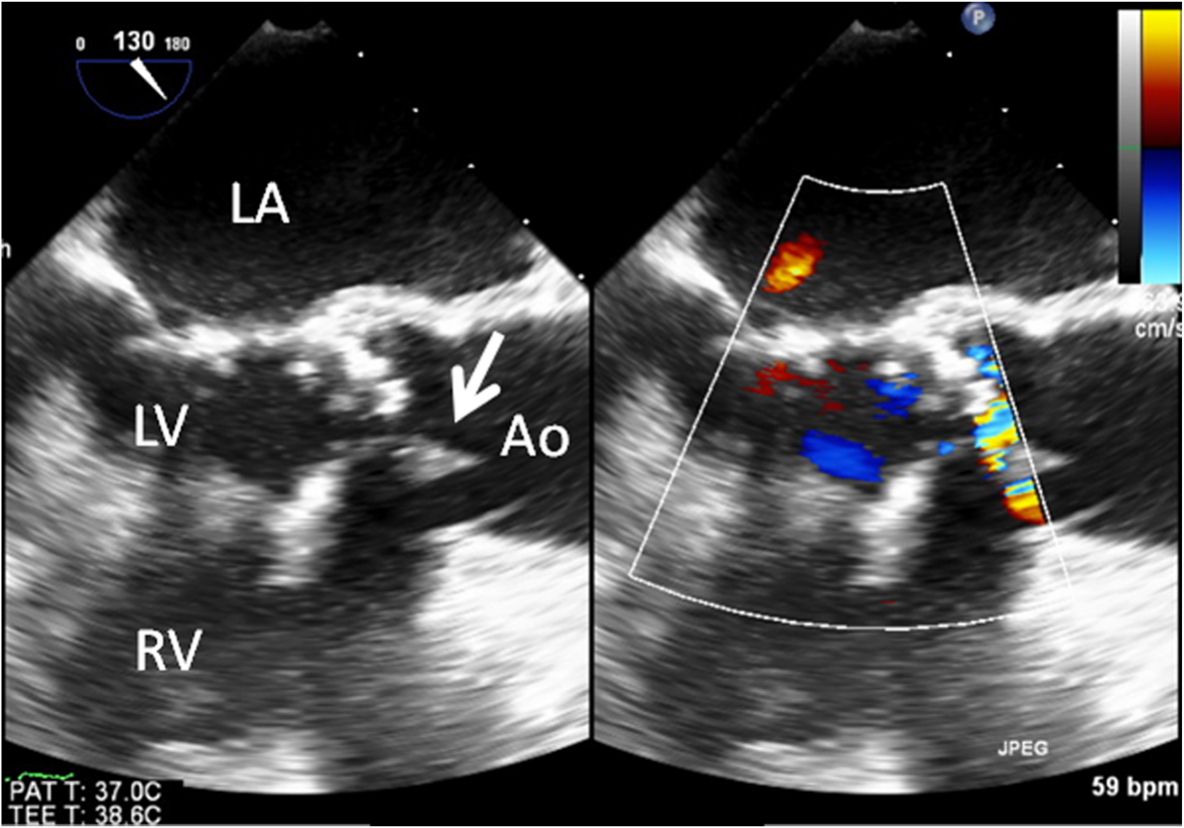
**Transesophageal echocardiogram.** A 12-mm diameter verruca was observed in the aortic valve at the non-coronary cusp-right coronary cusp (NCC–RCC) junction.

**Figure 2:**
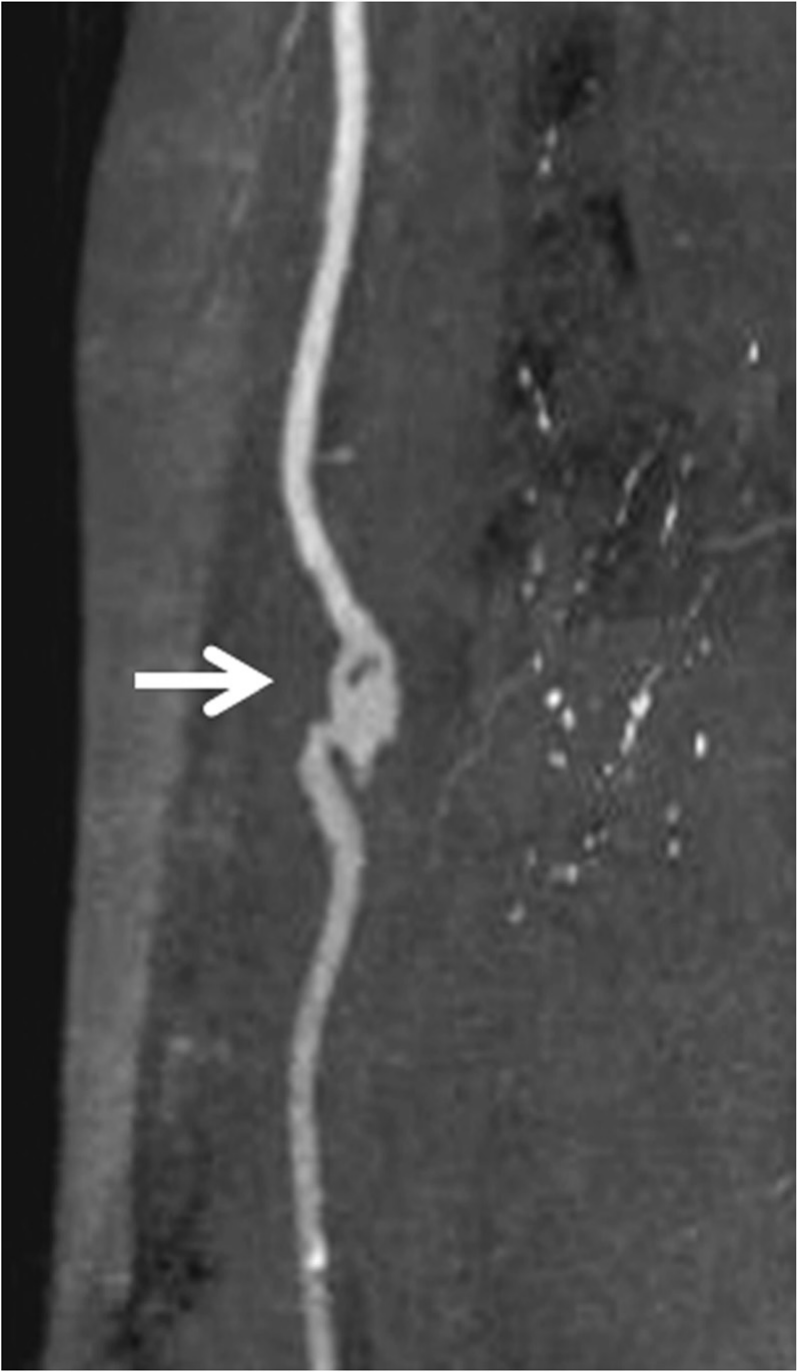
**Right brachial artery (three-dimensional computed tomography [3D-CT]).** This was believed to be an infected aneurysm associated with the dialysis procedure; the aneurysm was present on the central side of the dialysis puncture site. It was believed that this site was infected because the fever was initially limited to the day of dialysis.

**Figure 3:**
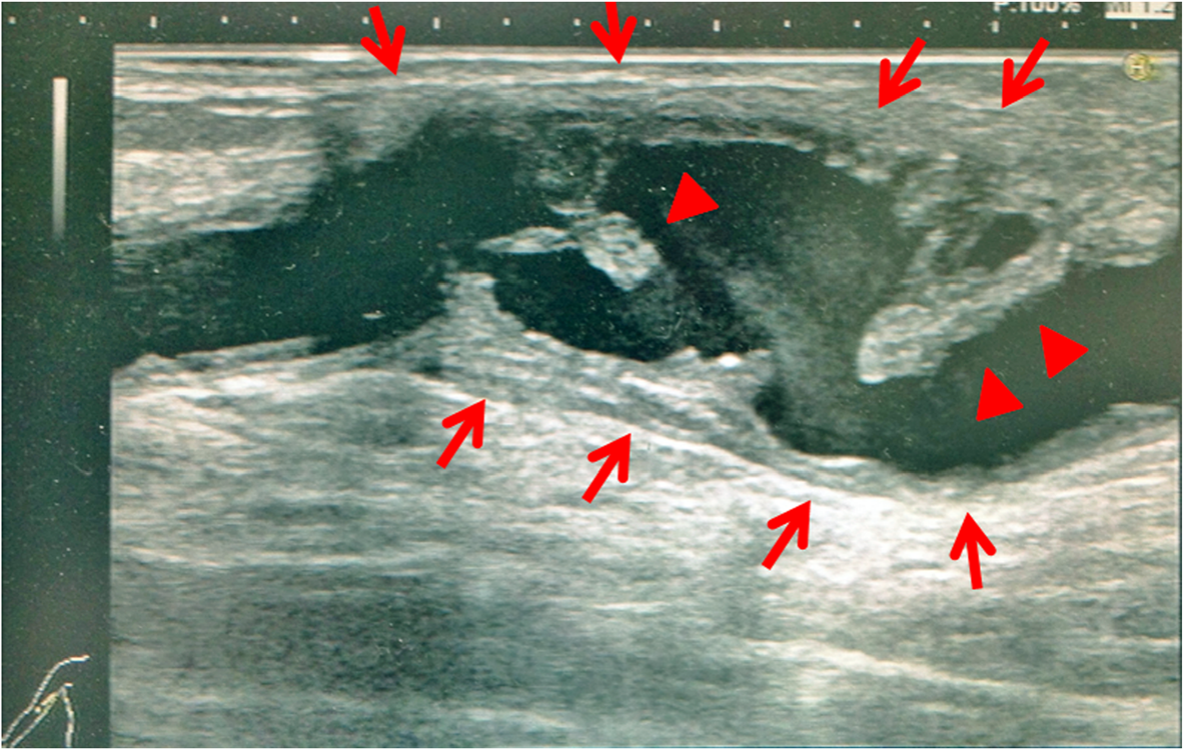
**Vascular echocardiogram of the right brachial artery.** An aneurysm was present on the central side of the dialysis puncture site. The aneurysm had irregular wall thickening (red arrows), and a protruding site was mobile owing to vascular pulsation (red arrowheads). This was in clear contrast to the wall thickening and arterial thrombus associated with arterial sclerosis, and it was believed to be a plaque (verruca) associated with an infection.

Chest-abdominal contrast CT and simple head MRI yielded no abnormal findings, suggesting no infection in particular. Transthoracic echocardiography was unable to point out any anomaly over than moderate aortic stenosis. Transesophageal echocardiography showed a 12-mm diameter verruca at the NCC-RCC junction of the aortic valve (Figure 1). Aneurysm and thrombus attachment appeared in three-dimensional-computerized tomography (3D-CT) and vascular echocardiography of the brachial artery that had been used as the puncture site during hemodialysis (Figures 2 and 3). The result of urine culture testing was negative. *E. durans* was detected in 7 sets of blood culture performed by the previous physician and at our hospital. Definitive identification of the isolate as *E. durans* was done by using the rapid streptococcus test (Rapid ID32 STREP; bioMerieux SA, Marcy l'Etoile, France) and confirmed by biochemical characterization, as recommended by Facklam & Collins (1989) [1] and Knudtson & Hartman (1992) [2]. These findings led to a diagnosis of subacute infective endocarditis and blood access infection/infective aneurysm caused by *E. durans*. Based on the results of antimicrobial susceptibility with *E. durans*, 2 g ampicillin I.V. every 24 h + 40 g/dose of gentamicin (0.8 mg/kg/dose) and post-dialysis I.V. (together with adjustment dosing during dialysis usage) were administered upon admission to our hospital. Drug susceptibility testing was carried out with highly concentrated gentamicin drug susceptibility testing using the disk method and the broth microdilution method from MicroScan WalkAway Pros Combo Panel 3.1 J (Siemens Healthcare Diagnostics K. K, Tokyo, Japan).

Given the patient's age and long history of diabetes, the patient's blood vessels had incurred significant arterial sclerosis. With a performance status of 3, surgical intervention was not performed, and therapy with antibiotics was the first choice. Antibiotic administration alleviated the fever, and subsequent blood culture testing showed negative results after 6 weeks of treatment. Transesophageal echocardiography performed during the course of treatment confirmed the disappearance of the verruca of the aortic valve. Vascular echocardiography of the brachial artery performed during the course of treatment revealed narrowing of the aneurysm. It also showed the disappearance of the thrombus, without the occurrence of any embolic symptoms. Antimicrobial therapy mitigated the condition, and the clinical diagnosis was a plaque (verruca) associated with an infection. Thus antibiotic administration mitigated the infective endocarditis and blood access infection/infective aneurysm and the patient was discharged. The patient has not relapsed after more than six months after discharge.

## Discussion

Among all Enterococcal infections, 63–81% are classified under *E. faecalis* and 13-23% are classified under *E. faecium*; the remainder is other species (*E. gallinarum, E. avium, E. casseliflavus, E. raffinosus, E.durans and E. hirae*, etc.) [3]. Following streptococci and staphylococci, the enterococci are 3rd most frequent of the bacteria causing infective endocarditis, accounting for about 8% [4]. However, *E. durans* infection is extremely rare, being reported in only 0.1% of *Enterococcal* bacteremia [5]. Moreover, worldwide, infective endocarditis caused by *E. durans* has been reported only in 3 cases ever [6-8]. In turn, being unable to find any reports of blood access infection or infective aneurysm caused by *E. durans*, we believe that this is the world's first report.*E. durans* infection is rare and sporadic in other organs, too, but reports do include urinary tract infection [9] and wound infection [3]. Also, *E. durans* infection has an easily followed sub-acute progression, and in the present case, the fever lasted for several months. Elderly and immunocompromised patients are more susceptible to Enterococcal infection [6]. Similarly, reports of *E. durans* infection are also found for immunocompromised patients with cirrhosis [6] or after renal transplantation [10], as well as complex infections preceded by some form of therapy [3,8]. In the present case, as well, the patient’s long-term history of hemodialysis is considered to have been a risk factor. Initially, infection was observed only after hemodialysis, so it is believed that blood access infection of the superficialized brachial artery occurred first, and was then complicated by infective endocarditis from bacteremia.

Transthoracic echocardiography failed to confirm the verruca because of its low sensitivity (43-60%) and the intensity of calcification of the aortic valve. However, transesophageal echocardiography has a sensitivity of 87-100% and specificity of 91-100% [11], and it was able to confirm the verruca of the aortic valve.

Optimal therapy for IE caused by enterococci requires a synergistic bactericidal combination of a cell-wall-active antimicrobial agent to which the organism is susceptible (penicillin or ampicillin) and an aminoglycoside. In the present case, the *E. durans* was favorably susceptible to ampicillin and gentamicin, and this concomitant antimicrobial therapy alone was able to improve the infective endocarditis and blood access infection.

## Conclusions

To conclude, we have experienced a case of concurrent infective endocarditis and blood access infection/infective aneurysm caused by *E. durans*. This is the world’s first reported case of blood access infection/infective aneurysm by *E. durans*.

## Consent

Written informed consent was obtained from the patient for publication of this Case report and any accompanying images.

A copy of the written consent is available for review by the Series Editor of this journal.

## Competing interests

All authors have no financial interests to disclose and no conflict of interest to declare.

## Authors’ contributions

TK: Manuscript redaction. NT: Management of the case, manuscript redaction and correction. AK: Clinical management of the case and relecture of the manuscript. KT: Manuscript correction, redaction of the comment of the illustrations. All authors read and approved the final manuscript.

## Pre-publication history

The pre-publication history for this paper can be accessed here:

http://www.biomedcentral.com/1471-2334/13/594/prepub
